# Selinexor in combination with venetoclax and decitabine in patients with refractory myelodysplastic syndrome previously exposed to hypomethylating agents: three case reports

**DOI:** 10.3389/fonc.2024.1477697

**Published:** 2024-12-19

**Authors:** Yunshuo Xiao, Kun Yang, Qiuying Huang, Changqing Wei, Manlv Wei, Zhili Geng, Hui Wu, Tianhong Zhou, Xialoin Yin, Yali Zhou

**Affiliations:** ^1^ Department of Hematology, The 923rd Hospital of the Joint Logistics Support Force of the People’s Liberation Army, Nanning, China; ^2^ Department of Hematology, Zigong First People’s Hospital, Zigong, China; ^3^ Department of Hematology, West China Hospital, Chengdu, China

**Keywords:** selinexor, venetoclax, hypomethylating agents, myelodysplastic syndrome, acute myeloid leukemia

## Abstract

The management of patients with myelodysplastic syndrome (MDS) refractory to hypomethylating agents (HMAs) remains a challenge with few reliably effective treatments. Preclinical studies have shown that the inhibition of the nuclear export protein XPO1 causes nuclear accumulation of p53 and disruption of NF-κB signaling; both of which are relevant targets for MDS. Selinexor is an XPO1 inhibitor with demonstrated efficacy in MDS patients. Herein, we report three patients with MDS refractory to HMAs, however, when selinexor and venetoclax were added to the treatment regimen, the patients achieved a complete response and a significant reduction in spleen size. All patients successfully underwent hematopoietic stem cell transplantation. These cases demonstrate that the combination therapy can achieve CR and significant reductions in spleen size, offering a promising therapeutic option for patients with limited treatment choices. Combination therapy would also offer a potential way for patients to bridge to transplantation. Formal evaluations of this regimen in patients with MDS refractory to HMAs may be meaningful.

## Introduction

Myelodysplastic syndrome (MDS) comprises a heterogeneous group of myeloid malignancies with distinct natural histories (1). For patients with high-risk MDS, there is no standard therapy for patients refractory to hypomethylating agents (HMAs) (2). The prognosis of patients with HMA failure is poor and overall survival in that setting is less than 6 months (3). Currently, there are no therapies with significant activities for this group of patients. For patients with higher risk HMA failure, options that have been investigated include rigosertib, venetoclax, and guadecitabine, among others (4, 5).

Exportin 1 (XPO1) is a protein that regulates the nuclear export of client proteins and has been found to play a critical role in multiple cancers (6). As a nuclear exporter, XPO1 plays a critical role in the intracellular localization of multiple proteins, as well as some mRNA transcripts. XPO1 is also required for the survival of solid tumors and hematological malignancies (7). Selinexor is a first-in-class selective inhibitor of nuclear export of compounds that inhibit XPO1 and has activity against hematologic and solid tumor malignancies. Selinexor inhibits XPO1 and has shown promising responses in multiple myeloma (8), acute myeloid leukemia (AML) (9, 10), and non-Hodgkin lymphoma (11) patients. Furthermore, Selinexor has shown efficacy in patients who fail to respond to HMA therapy (12). Previous attempts to treat patients with MDS resistant to HMAs have failed to achieve efficacy and safety results. Studies have shown that selinexor has synergistic effects with HMAs and B-cell lymphoma-2 (BCL-2) inhibitors, while its application can enhance the killing of tumor cells (13, 14). Thus, we assessed a three-drug combination therapy in this study. Here, we present three refractory MDS patients who were unresponsive to azacitidine, including the venetoclax plus azacitidine regimen, but were successfully treated by selinexor in combination with venetoclax and decitabine. The regimen was safe and effective, and may provide a new option for relapsed or refractory MDS patients with resistance to HMAs.

## Case presentations

### Patient 1

A 35-year-old female was diagnosed with MDS with anemia in September 2020. Bone marrow (BM) aspiration revealed a hypercellular BM with 2% blasts and erythrophyletic, granulopathic, and megakaryotic pathological hematopoiesis. Cytogenetics and fluorescence *in situ* hybridization were normal. Initially, the patient was treated with cyclosporine and blood transfusion support. During that period, the patient also received four cycles of azacitidine and achieved hematological improvement. In April 2022, the patient’s anemia worsened. Bone marrow aspiration revealed a hypercellular BM with 2.5% blasts and trilineage pathological hematopoiesis. Bone marrow biopsy revealed marrow fibrosis-1 (MF-1). Chromosomal analysis revealed a normal karyotype. Myeloid tumor-related gene detection showed that the patient was positive for *ASXL1*, *STAG2*, and *TET2*. Fusion genes were all negative. Hematopoietic stem cell transplantation (HSCT) was recommended, which the patient refused. The patient continued to receive azacitidine for seven cycles and the disease progressed. Repeat bone marrow cytology showed 9% blasts. Bone marrow biopsy revealed MF-2. Myeloid tumor-related gene detection revealed positivity for *ASXL1*, *BCOR*, *CEBPA*, *CRS3R*, *IDH2*, *STAG2*, and *TET2*. Ultrasonography revealed splenomegaly of 19.6 cm long and 4.7 cm thick. Therefore, MDS with secondary myelofibrosis was diagnosed.

The patient was then treated with decitabine combined with venetoclax and selinexor [decitabine once daily (10mg day1-5), venetoclax once daily (100 mg day1-21), and selinexor twice per week (40mg day2, 5, 9, 12, 16, 19)] as a salvage therapy. complete remission (CR) was achieved after one cycle of therapy, and the bone marrow blast counts decreased from 9% to 0%. The spleen size was significantly reduced to 15.2 cm long and 4.0 cm thick. The adverse events (AEs) occurring during treatment were mainly grade 1-2 nausea and hematological toxicity. Self-improvement was achieved without special treatment. The patient then completed allogeneic HSCT. The complete treatment process is shown in **Table 1**. The patient remains on maintenance treatment with decitabine, and currently experiences CR and minimal residual disease is negative based on flow cytometry. As of November 2024, the patient had a recurrence-free survival for 9 months.

**Table 1:** Clinical characteristics and complete treatment process of the three patients.

	Case1	Case2	Case3
Age/Sex	36/Female	39/female	48/Male
Height/Weight	157cm/46kg	157cm/42kg	170cm/56kg
morphologically defined	MDS-LB	MDS-IB1	MDS-h
Cytogenetics	46,XX	45~46,XX,-4,-5,add(5)(q22),del(6)(p21),add(7)(q11),?del(16)(q12),add(19)(p13),add(10)(p13),+mar1,+mar2,Idmin[cp16]/46,XX[4]	46,XY,+1,der(1:16)(q10:p10)[12]/46,XY[8]
Molecular biology	ASXL1, BCOR, CEBPA, CRS3R, IDH2, STAG2, TET2	DNMT3A, SH2B3, TP53	ASXL1, CSF3R, E2AF1, TP53
Leukemic Transformation	MDS	MDS-to-AML	MDS-to-AML
MF grading	MF-2	MF-2	MF-1
Previous treatment	azacytidine, ruxolitinib	azacytidine, decitabine, venetoclax	azacytidine, decitabine
Spleen size	19.6cm length, 4.7cm thickness, 9.5cm below costal margin	20.2cm length, 7.1cm thickness, 8.5cm below costal margin	17cm length, 4.9cm thickness, 5cm below costal margin
SVD treatment	1 cycle	2 cycles	1 cycle
Pre-transplant spleen size	14.5cm length, 4.2cm thickness, 2.5cm below costal margin	14.6cm length, 4.8cm thickness, 2.3cm below costal margin	15.2cm length, 4.0cm thickness, 2cm below costal margin
Pre-transplant response^#^	CRh	NA^§^	CRi
Donor	HLA 6/10-matched brother	HLA fully-matched brother	HLA fully-matched brother
Pre-transplantation regimen	fludarabine 30mg/m^2^ -7~-2 days, busulfan 130mg/m^2^ -7~-6 days, melphalan 100mg/m^2^ -5 day	fludarabine 30mg/m^2^ -7~-2 days, busulfan 130mg/m^2^ -7~-6 days, melphalan 100mg/m^2^ -4 day, thiotepa 5mg/kg -5 day	fludarabine 30mg/m^2^ -7~-2 days, busulfan 130mg/m^2^ -7~-6 days, melphalan 100mg/m^2^ -5 day
GVHD prevention	PTCY +3d, +4d 25mg/kg/d ATG +5d 25mg/kg/d Cyclosporine	PTCY +3d, +4d 25mg/kg/d Cyclosporine	PTCY +3d, +4d 25mg/kg/d Cyclosporine
Neutrophil engraftment	13d	14d	15d
platelet engraftment	14d	19d	17d
Maintenance therapy	decitabine 10mg for 3 days every 4 weeks	decitabine 10mg for 3 days every 4 weeks	decitabine 10mg for 3 days every 4 weeks
Survival status after transplantation	RFS for 9 months	died 4 months after transplantation	RFS for 7 months

MDS, myelodysplastic syndrome; AML, acute myeloid leukemia; MF, myelofibrosis; SVD, selinexor, venetoclax, decitabine; HLA, human leukocyte antigen; GVHD, graft versus host disease; PTCY, post-transplant cyclophosphamide; ATG, Anti-human T lymphocyte porcine immunoglobulin; RFS, recurrence-free survival.

^#^Response criteria according to the 2022 editions of the European LeukemiaNet.

^§^The aplastic state does not meet the assessment criteria.

### Patient 2

A 39-year-old female was diagnosed with MDS in July 2023 with pancytopenia. Bone marrow aspiration revealed a hypercellular BM with 8% blasts and erythroid and megakaryocyte pathological hematopoiesis. Cytogenetics revealed a complex karyotype, and a molecular panel identified aberrations in *DNMT3A*, *SH2B3*, and *TP53*. Fusion genes were all negative. Fluorescence *in situ* hybridization revealed a 17p deletion. The patient was treated with azacitidine for two cycles, but progressive disease was observed. Bone marrow examination showed a hypercellular marrow with 34% myeloid blasts. Bone marrow biopsy revealed MF-2. Ultrasonography showed splenomegaly of 20.2 cm long and 7.1 cm thick. According to the guidelines, AML can be diagnosed when the number of bone marrow blast counts in MDS patients increases to more than 20% (15). Therefore, acute myeloid leukemia transformed by MDS was diagnosed. The patient received one cycle of treatment with azacitidine combined with venetoclax [azacitidine once daily (100 mg, days 1-7); venetoclax once daily (100 mg, days 1-14)], however, bone marrow did not exhibit remission. Then, decitabine combined with venetoclax and selinexor [decitabine once daily (10 mg, days 1-5), venetoclax once daily (100 mg, days 1-21), and selinexor three times per week (20 mg, days 2, 4, 6, 9, 11, 13, 16, 18, 20)] was administered. CR was achieved after one cycle of therapy, and the bone marrow blast count was decreased from 34% to 3%. The spleen size was significantly reduced to 12.1 cm long and 4.0 cm thick. The AEs occurring during treatment were mainly grade 1-2 nausea and hematological toxicity. Self-improvement was achieved without special treatment. The patient then completed allogeneic HSCT. The complete treatment process is shown in **Table 1**. After one cycle of decitabine maintenance therapy, the patient unfortunately passed away due to relapse.

### Patient 3

A 48-year-old male was diagnosed with MDS in April 2020 with pancytopenia. Bone marrow aspiration revealed megakaryocyte pathological hematopoiesis. Flow cytometry showed the immunophenotype with 3% myeloid blasts. Chromosomal analysis revealed a normal karyotype. After the diagnosis of MDS, the patient was initiated on azacitidine treatment and was evaluated several times, during which the disease was stable. However, the patient’s disease progressed in February 2024. Bone marrow examination revealed hypercellular marrow with 36% myeloid blasts. Flow cytometry analysis revealed the immunophenotype of myeloid blasts. Cytogenetics analysis revealed a karyotype of 46,XY,+1,der(1:16)(q10:p10)[12]/46,XY[8], and a molecular panel identified aberrations in *ASXL1, CSF3R, E2AF1*, and *TP53*. Fusion genes were all negative. Ultrasonography revealed splenomegaly of 17.0 cm long and 4.9 cm thick. As bone marrow blast counts increased to >20%, AML transformed by MDS was diagnosed. Decitabine combined with venetoclax and selinexor [decitabine once daily (10 mg, days 1-5), venetoclax once daily (100 mg, days 1-21), and selinexor three times per week (20 mg, days 2, 4, 6, 9, 11, 13, 16, 18, 20)] was then administered. CR was achieved after one cycle of therapy, and the bone marrow blast counts were decreased from 36% to 0%. The spleen was also significantly reduced in size to 15.2 cm long and 4.0 cm thick. The AEs occurring during treatment were mainly grade 1-2 nausea and hematological toxicity. Self-improvement was achieved without special treatment. The patient then completed allogeneic HSCT. The complete treatment process is shown in **Table 1**. The patient is currently receiving maintenance treatment with decitabine, and is experiencing CR. Minimal residual disease is negative based on flow cytometry. As of November 2024, the patient had a recurrence-free survival of 7 months.

## Discussion

How to overcome the poor prognosis of patients with MDS resistant to HMAs is a challenge for clinicians. Prebet et al. (3) compared the prognosis of patients undergoing different treatments after the failure of azacitidine therapy, and found the following (from best to worst): allogeneic HSCT, experimental therapy, traditional chemotherapy, and supportive therapy. Rigosertib targets the Ras binding regions of different kinases such as RAF and PI3K, thereby inhibiting related pathways and stopping cell division and promoting apoptosis of tumor cells. In phase III clinical trials, MDS patients with HMA treatment failure had a median survival of 8.2 months following Rigosertib treatment, which was not significantly different than 5.9 months for the controls (5). Histone deacetylase inhibitors inhibit DNA transcription by removing the acetyl group of lysine from histones. Histone deacetylase inhibitors have been used in multiple clinical trials in MDS patients who have failed on HMAs, but it is ineffective, with a low response rate. Moreover, combination therapies with HMAs may increase the incidence of adverse effects (16). In MDS patients refractory to HMAs, the conventional AML chemotherapy regimen has an effective response rate of 14% to 30% (17). New chemotherapy regimens may be more efficient, such as the clofarabine plus cytarabine regimen, which was 44% effective in 70 MDS patients with HMA treatment failure. Those patients also had an overall median survival of 10 months, and a median survival of 22 months. Multivariate analysis suggests that a complex karyotype is less likely to be effective for this treatment (18). Given the unsatisfactory clinical trial results above, additional exploration is needed to identify an effective and safe treatment option.

Selinexor is an XPO1 inhibitor. XPO1 is a major nuclear export protein that transports a variety of proteins and some nuclear RNA out of the nucleus through the nuclear pore complex embedded in the nuclear membrane. XPO1 can thus regulate cell proliferation and apoptosis through the nucleocytoplasmic transport mechanism. In hematological malignancies, XPO-1 is often overexpressed. Malignant tumor cells that overexpress XPO-1 transport tumor suppressor proteins, such as p53, cell cycle proteins, and oncoproteins to the cytoplasm. Tumor suppressor cells are unable to recognize and kill tumor cells, and immune response regulatory proteins and oncoproteins also become abnormal, further leading to abnormal regulation of cell growth and apoptosis, or abnormal cell cycle (19). In addition, XPO overexpression is associated with multiple tumor resistance to anthracyclines, tyrosine kinase inhibitors, and platinum compounds, among others (19).

NF-κB is a transcription factor that is involved in the production of inflammatory factors and regulates cell apoptosis, proliferation, and differentiation. NF-κB is activated in the stem cells of MDS patients, leading to impaired function of hematopoietic stem and progenitor cells, which is associated with increased ferritin levels, increased proportion of primitive cells, increased IL-8 levels, and inflammatory microenvironments (20). TP53 is a common non-driver mutation in MDS patients and an important indicator of poor prognosis. TP53 mutation will lead to an overexpression of p53 protein and affect the progression of disease (21). P53 regulates the expression of downstream target genes and is involved in multiple cellular processes. Abnormalities in p53 function are associated with increased disease risk, rapid conversion to acute myeloid leukemia, and drug resistance in MDS patients (22–24). One of the main reasons that p53 performs its normal function is because of its intracellular localization. P53 mainly plays a tumor suppressor function in the nucleus, and is inactivated when translocated to the cytoplasm. Cancer cells use the nuclear cytoplasm through the transport of nuclear pore complexes to stimulate tumor growth while promoting tumor immune escape. In this regard, targeted nuclear export inhibition becomes a potential target for therapeutic intervention in cancer (25, 26). In normal cells, p53 is strictly regulated, which monitors DNA damage repair in the nucleus, and can also trigger cell cycle arrest or induce apoptotic effects by promoting the transcription of p21. However, XPO1-mediated excessive export of p53 to the cytoplasm is observed in many tumor cells, leading to its loss of function. Selinexor can highly selectively act on XPO1 targets, modify the abnormal localization of p53 proteins inside and outside the nucleus, promote the nuclear aggregation of p53, p21 and p73, and exert antitumor effects. At the same time, selinexor can also upregulate the level of p53 downstream signal BAX, PUMA, thus promoting apoptosis (27–29). Preclinical studies have shown that inhibition of XPO-1 can lead to p53 nuclear accumulation and blockade of the NF-κB signaling pathway, both of which are therapeutic targets in MDS (30, 31).

In an investigator-initiated, single-institution, single-arm phase II study, the investigators included a total of 25 patients with refractory MDS and oligoblastic AML who did not respond to HMA therapy. Over the course of the study, six patients achieved marrow complete response, 12 patients were stable, and the median overall survival of all patients was 8.5 months, with a 1-year survival rate of 28% (12). Additionally, Eltanexor is a second-generation selective inhibitor of nuclear export, which has lower blood-brain barrier penetration and a wider therapeutic window than selinexor. In high-risk myelodysplastic syndromes, eltanexor induced a total disease control rate of 53.3% (32). Thus, XPO-1 inhibitors confer clinical benefit in high-risk MDS patients who do not respond to HMA therapy.

Evidence is emerging on the synergistic effects of selinexor in patients with hematological malignancies. *In vitro* investigations of AML cell lines and primary blasts demonstrated that the effectiveness of decitabine was substantially enhanced when combined with selinexor (14). Venetoclax is an oral BCL-2 inhibitor that has been used in combination with HMA for the treatment of MDS, and has exhibited high overall response rates and prolonged survival. However, primary and acquired resistance to venetoclax-based regimens remains a major concern (33, 34). As venetoclax resistance may be related to increased MCL-1 expression, selinexor, or its newer derivative—which reduces MCL-1 through the inhibition of its mRNA transport into the cytoplasm—may be rational drugs to combine with venetoclax (35). Selinexor has been verified to synergize with venetoclax to induce apoptosis in AML cells through the down‐regulation of MCL‐1 (35). Selinexor and eltanexor have also been shown to synergize with the BCL-2 inhibitor venetoclax in double hit lymphoma cell lines and patient-derived xenografts harboring MYC and BCL-2 alterations (36).

For the three MDS patients resistant to HMAs, the decitabine combined with venetoclax and selinexor regimen achieved rapid remission. During the course of treatment, we were surprised to find that the effect of spleen shrinkage was significant, which achieved the transplantation conditions and bridged the allogeneic hematopoietic stem cell transplantation. Patient 3 in this report did not respond well to the addition of venetoclax plus azacitidine at the time of disease progression, but CR was achieved after continued treatment with selinexor. To some extent, those findings demonstrated the synergistic effect of selinexor with HMAs and BCL-2 inhibitors. Secondary acute myeloid leukemia (sAML) occurs more frequently in elderly patients, and is associated with adverse cytogenetics and a multidrug resistance phenotype, which leads to poor prognosis. As a result, AML patients with a prior history of MDS experience lower CR rates (37). Two patients were converted to sAML, and rapid CR was achieved after the application of decitabine combined with venetoclax and selinexor regimen, thus, indicating that this treatment regimen has potential application for sAML and can overcome the poor prognosis of sAML.

## Conclusion

This study suggests that the combination of Selinexor, venetoclax, and decitabine is a viable option for patients with refractory MDS who were previously exposed to HMA. Selinexor is a promising therapeutic agent for the management of MDS and may offer encouraging results in combination with other drugs, such as venetoclax and HMA. Combination therapy offers a potential way for patients to bridge to transplantation. Investigating this combination regimen in refractory MDS patients may be meaningful in future planned trials. As this is a retrospective study based on three cases that lacked control groups and randomization, the findings have limited generalizability. In the future, multicenter randomized controlled trials will be required to verify the efficacy of combination therapies and clarify the impact of patient subgroup characteristics on treatment.

## Data Availability

The original contributions presented in the study are included in the article/supplementary material. Further inquiries can be directed to the corresponding author.

## References

[1] Khoury JD Solary E Abla O Akkari Y Alaggio R Apperley JF. The 5th edition of the World Health Organization classification of haematolymphoid tumours: myeloid and histiocytic/dendritic neoplasms. Leukemia. (2022) 36:1703–19. doi: 10.1038/s41375-022-01613-1 PMC9252913 35732831

[2] Garcia-Manero G. Myelodysplastic syndromes: 2023 update on diagnosis, risk-stratification, and management. Am J Hematol. (2023) 98:1307–25. doi: 10.1002/ajh.26984 PMC12002404 37288607

[3] Prebet T Gore SD Esterni B Gardin C Itzykson R Thepot S. Outcome of high-risk myelodysplastic syndrome after azacitidine treatment failure. J Clin Oncol. (2011) 29:3322–7. doi: 10.1200/JCO.2011.35.8135 PMC4859209 21788559

[4] Bazinet A Darbaniyan F Jabbour E Montalban-Bravo G Ohanian M Chien K. Azacitidine plus venetoclax in patients with high-risk myelodysplastic syndromes or chronic myelomonocytic leukaemia: phase 1 results of a single-centre, dose-escalation, dose-expansion, phase 1-2 study. Lancet Haematol. (2022) 9:e756–e65. doi: 10.1016/S2352-3026(22)00216-2 36063832

[5] Garcia-Manero G Fenaux P Al-Kali A Baer MR Sekeres MA Roboz GJ. Rigosertib versus best supportive care for patients with high-risk myelodysplastic syndromes after failure of hypomethylating drugs (Ontime): A randomised, controlled, phase 3 trial. Lancet Oncol. (2016) 17:496–508. doi: 10.1016/S1470-2045(16)00009-7 26968357

[6] Turner JG Dawson J Sullivan DM. Nuclear export of proteins and drug resistance in cancer. Biochem Pharmacol. (2012) 83:1021–32. doi: 10.1016/j.bcp.2011.12.016 PMC4521586 22209898

[7] Ishizawa J Kojima K Hail N Jr. Tabe Y Andreeff M. Expression, function, and targeting of the nuclear exporter chromosome region maintenance 1 (Crm1) protein. Pharmacol Ther. (2015) 153:25–35. doi: 10.1016/j.pharmthera.2015.06.001 26048327 PMC4526315

[8] Chen C Siegel D Gutierrez M Jacoby M Hofmeister CC Gabrail N. Safety and efficacy of selinexor in relapsed or refractory multiple myeloma and waldenstrom macroglobulinemia. Blood. (2018) 131:855–63. doi: 10.1182/blood-2017-08-797886 29203585

[9] Garzon R Savona M Baz R Andreeff M Gabrail N Gutierrez M. A phase 1 clinical trial of single-agent selinexor in acute myeloid leukemia. Blood. (2017) 129:3165–74. doi: 10.1182/blood-2016-11-750158 PMC5524530 28336527

[10] Wang AY Weiner H Green M Chang H Fulton N Larson RA. A phase I study of selinexor in combination with high-dose cytarabine and mitoxantrone for remission induction in patients with acute myeloid leukemia. J Hematol Oncol. (2018) 11:4. doi: 10.1186/s13045-017-0550-8 29304833 PMC5756334

[11] Kuruvilla J Savona M Baz R Mau-Sorensen PM Gabrail N Garzon R. Selective inhibition of nuclear export with selinexor in patients with non-Hodgkin lymphoma. Blood. (2017) 129:3175–83. doi: 10.1182/blood-2016-11-750174 28468797

[12] Taylor J Mi X Penson AV Paffenholz SV Alvarez K Sigler A. Safety and activity of selinexor in patients with myelodysplastic syndromes or oligoblastic acute myeloid leukaemia refractory to hypomethylating agents: A single-centre, single-arm, phase 2 trial. Lancet Haematol. (2020) 7:e566–e74. doi: 10.1016/S2352-3026(20)30209-X PMC9209897 32735836

[13] Fischer MA Friedlander SY Arrate MP Chang H Gorska AE Fuller LD. Venetoclax response is enhanced by selective inhibitor of nuclear export compounds in hematologic Malignancies. Blood Adv. (2020) 4:586–98. doi: 10.1182/bloodadvances.2019000359 PMC7013257 32045477

[14] Ranganathan P Yu X Santhanam R Hofstetter J Walker A Walsh K. Decitabine priming enhances the antileukemic effects of exportin 1 (Xpo1) selective inhibitor selinexor in acute myeloid leukemia. Blood. (2015) 125:2689–92. doi: 10.1182/blood-2014-10-607648 PMC4408293 25716206

[15] Dohner H Wei AH Appelbaum FR Craddock C DiNardo CD Dombret H. Diagnosis and management of Aml in adults: 2022 recommendations from an international expert panel on behalf of the eln. Blood. (2022) 140:1345–77. doi: 10.1182/blood.2022016867 35797463

[16] Prebet T Sun Z Figueroa ME Ketterling R Melnick A Greenberg PL. Prolonged administration of azacitidine with or without entinostat for myelodysplastic syndrome and acute myeloid leukemia with myelodysplasia-related changes: results of the us leukemia intergroup trial E1905. J Clin Oncol. (2014) 32:1242–8. doi: 10.1200/JCO.2013.50.3102 PMC3986386 24663049

[17] Zeidan AM Kharfan-Dabaja MA Komrokji RS. Beyond hypomethylating agents failure in patients with myelodysplastic syndromes. Curr Opin Hematol. (2014) 21:123–30. doi: 10.1097/MOH.0000000000000016 PMC4124617 24335709

[18] Jabbour E Faderl S Sasaki K Kadia T Daver N Pemmaraju N. Phase 2 study of low-dose clofarabine plus cytarabine for patients with higher-risk myelodysplastic syndrome who have relapsed or are refractory to hypomethylating agents. Cancer. (2017) 123:629–37. doi: 10.1002/cncr.30383 PMC11770834 27741352

[19] Azmi AS Uddin MH Mohammad RM. The nuclear export protein Xpo1 - from biology to targeted therapy. Nat Rev Clin Oncol. (2021) 18:152–69. doi: 10.1038/s41571-020-00442-4 33173198

[20] Vegivinti CTR Keesari PR Veeraballi S Martins Maia CMP Mehta AK Lavu RR. Role of innate immunological/inflammatory pathways in myelodysplastic syndromes and Aml: A narrative review. Exp Hematol Oncol. (2023) 12:60. doi: 10.1186/s40164-023-00422-1 37422676 PMC10329313

[21] Zhang L McGraw KL Sallman DA List AF. The role of P53 in myelodysplastic syndromes and acute myeloid leukemia: molecular aspects and clinical implications. Leuk Lymphoma. (2017) 58:1777–90. doi: 10.1080/10428194.2016.1266625 27967292

[22] Haase D Stevenson KE Neuberg D Maciejewski JP Nazha A Sekeres MA. Tp53 mutation status divides myelodysplastic syndromes with complex karyotypes into distinct prognostic subgroups. Leukemia. (2019) 33:1747–58. doi: 10.1038/s41375-018-0351-2 PMC6609480 30635634

[23] Kitagawa M Yoshida S Kuwata T Tanizawa T Kamiyama R. P53 expression in myeloid cells of myelodysplastic syndromes. Association with evolution of overt leukemia. Am J Pathol. (1994) 145:338–44. doi: 10.1111/j.1399-0039.1994.tb02382.x PMC1887403 8053492

[24] Lindsley RC Saber W Mar BG Redd R Wang T Haagenson MD. Prognostic mutations in myelodysplastic syndrome after stem-cell transplantation. N Engl J Med. (2017) 376:536–47. doi: 10.1056/NEJMoa1611604 PMC5438571 28177873

[25] Tan DS Bedard PL Kuruvilla J Siu LL Razak AR. Promising sines for embargoing nuclear-cytoplasmic export as an anticancer strategy. Cancer Discovery. (2014) 4:527–37. doi: 10.1158/2159-8290.CD-13-1005 24743138

[26] Tran EJ King MC Corbett AH. Macromolecular transport between the nucleus and the cytoplasm: advances in mechanism and emerging links to disease. Biochim Biophys Acta. (2014) 1843:2784–95. doi: 10.1016/j.bbamcr.2014.08.003 PMC4161953 25116306

[27] Gupta A Saltarski JM White MA Scaglioni PP Gerber DE. Therapeutic targeting of nuclear export inhibition in lung cancer. J Thorac Oncol. (2017) 12:1446–50. doi: 10.1016/j.jtho.2017.06.013 PMC5572747 28647672

[28] Nachmias B Schimmer AD. Targeting nuclear import and export in hematological Malignancies. Leukemia. (2020) 34:2875–86. doi: 10.1038/s41375-020-0958-y PMC7584478 32624581

[29] Tai YT Landesman Y Acharya C Calle Y Zhong MY Cea M. Crm1 inhibition induces tumor cell cytotoxicity and impairs osteoclastogenesis in multiple myeloma: molecular mechanisms and therapeutic implications. Leukemia. (2014) 28:155–65. doi: 10.1038/leu.2013.115 PMC3883926 23588715

[30] Yu L Mohamed AJ Simonson OE Vargas L Blomberg KE Bjorkstrand B. Proteasome-dependent autoregulation of Bruton tyrosine kinase (Btk) promoter via Nf-Kappab. Blood. (2008) 111:4617–26. doi: 10.1182/blood-2007-10-121137 18292289

[31] David-Watine B. Silencing nuclear pore protein Tpr elicits a senescent-like phenotype in cancer cells. PloS One. (2011) 6:e22423. doi: 10.1371/journal.pone.0022423 21811608 PMC3139644

[32] Lee S Mohan S Knupp J Chamoun K de Jonge A Yang F. Oral eltanexor treatment of patients with higher-risk myelodysplastic syndrome refractory to hypomethylating agents. J Hematol Oncol. (2022) 15:103. doi: 10.1186/s13045-022-01319-y 35922861 PMC9351096

[33] Ball BJ Famulare CA Stein EM Tallman MS Derkach A Roshal M. Venetoclax and hypomethylating agents (Hmas) induce high response rates in Mds, including patients after Hma therapy failure. Blood Adv. (2020) 4:2866–70. doi: 10.1182/bloodadvances.2020001482 PMC7362378 32589727

[34] Azizi A Ediriwickrema A Dutta R Patel SA Shomali W Medeiros B. Venetoclax and hypomethylating agent therapy in high risk myelodysplastic syndromes: A retrospective evaluation of a real-world experience. Leuk Lymphoma. (2020) 61:2700–7. doi: 10.1080/10428194.2020.1775214 32543932

[35] Luedtke DA Su Y Liu S Edwards H Wang Y Lin H. Inhibition of Xpo1 enhances cell death induced by Abt-199 in acute myeloid leukaemia via Mcl-1. J Cell Mol Med. (2018) 22:6099–111. doi: 10.1111/jcmm.13886 PMC6237582 30596398

[36] Liu Y Azizian NG Dou Y Pham LV Li Y. Simultaneous targeting of Xpo1 and Bcl2 as an effective treatment strategy for double-hit lymphoma. J Hematol Oncol. (2019) 12:119. doi: 10.1186/s13045-019-0803-9 31752970 PMC6868798

[37] Ye XN Zhou XP Wei JY Xu GX Li Y Mao LP. Epigenetic priming with decitabine followed by low-dose idarubicin/cytarabine has an increased anti-leukemic effect compared to traditional chemotherapy in high-risk myeloid neoplasms. Leuk Lymphoma. (2016) 57:1311–8. doi: 10.3109/10428194.2015.1091931 26372888

